# Analysis of Road Network Pattern Considering Population Distribution and Central Business District

**DOI:** 10.1371/journal.pone.0151676

**Published:** 2016-03-16

**Authors:** Fangxia Zhao, Huijun Sun, Jianjun Wu, Ziyou Gao, Ronghui Liu

**Affiliations:** 1 State Key Laboratory of Rail Traffic Control and Safety, Beijing Jiaotong University, Beijing, China; 2 School of Traffic and Transportation, Beijing Jiaotong University, Beijing, China; 3 Institute for Transport Studies, University of Leeds, Leeds LS2 9JT, United Kingdom; Beihang University, CHINA

## Abstract

This paper proposes a road network growing model with the consideration of population distribution and central business district (CBD) attraction. In the model, the relative neighborhood graph (RNG) is introduced as the connection mechanism to capture the characteristics of road network topology. The simulation experiment is set up to illustrate the effects of population distribution and CBD attraction on the characteristics of road network. Moreover, several topological attributes of road network is evaluated by using coverage, circuitness, treeness and total length in the experiment. Finally, the suggested model is verified in the simulation of China and Beijing Highway networks.

## 1. Introduction

Road network pattern is an important problem in the transportation science which has long been recognized as a key component for Network design problem (NDP). For the detailed reviews of NDP, the readers are referred to [1–10]. In fact, the road network pattern problem determines the distribution of the various elements of road network in the space [11–16]. In reality, the transportation planners generally focus on that which pattern of urban road network is better for a city. This because that the rational plan and layout of road network pattern can not only reduce traffic incidence of the traffic jam and produce spatial agglomeration economy [17, 18], but also save the investment cost. Therefore, the road network pattern problem has become one of the most active research fields in transportation science in the past decades. According to the view of geography, road network pattern can be investigated from human and urban physical aspects.

From the human aspect, road network pattern appears the spatial structure of population distribution. Recently, a variety of papers have focused on the road network pattern from human aspect [19–22]. For example, Yamins et al. [19] proposed a co-evolution model between population distribution and road system, which can generate global features as belt-ways and star patterns observed in urban transportation infrastructure. Barthélemy and Flammini [20] developed a basic model that describes the impact of economical mechanisms on the evolution of the population distribution and the topology of the road network. Garcia-López [21] presented the effect of transportation improvements on changes in population location patterns in metropolitan Barcelona between 1991 and 2006.

From the urban physical aspect, it is hard to image that the deterioration of traffic congestion and air is more likely to produce in the densely population. To alleviate the traffic jam and the air pollution, the concept of multi-CBD road network pattern has been accepted by more and more scholars, and gradually has become the trend of the road network pattern. Recently, there is an increasing body of relevant literature on the impact of multi-CBD on the topology of road network. For example, Lin et al. [23] developed the evolution of road network of Beijing CBD in 2020 using simulation software VISSIM. Buckwaler [24] analyzed the impact of multi-CBD on the road-building and network efficiency in Pittsburgh 1980–2010. Huang and Wei [25] revealed the CBDs have positive impacts on the region spatial structure. In this paper, the impact of population distribution and multi-CBD on the topology of road network is simultaneously considered.

Generally speaking, the road network pattern model indeed follows a simple universal mechanism [26–30], such as minimum spanning tree (MST) or shortest path tree. In fact, it is natural to image that minimum spanning tree can be used as network connecting mechanism in the study of road network pattern since its connectivity. Generally, the accessibility of road network is low for the minimum spanning tree. Additionally, the construction cost of road based on MST is always not the minimal [31]. Therefore, to minimize the road construction cost, minimum Steiner tree [31] is also adopted as the network connecting mechanism. Minimum Steiner tree is a graph that connects the known points by lines of minimum total length in such a way that any two points may be interconnected by line segments either directly or via the new added points [32]. However, the limitation in the application of minimum Steiner tree to the study of urban road pattern with the consideration of population distribution is that the weight of each zone is assumed to be equal. In reality, the population of each zone is not same. Therefore, the impact of different zone on the urban road network pattern is not same, which implies that the weight of each zone should not be the same. Therefore, to improve the accessibility of network and to avoid too many cyclical paths in the network, in this paper, relative neighborhood graph [31] is adopted as network connecting mechanism due to its good accessibility [33]. RNG is an undirected graph defined on a set of zones in the Euclidean plane by connecting two distinct zones and by an edge whenever there does not exist a third zone that is closer to both than they are to each other. In fact, MST is a subgraph of RNG, which means that RNG has higher accessibility and smaller number of circles in the network than MST [33]. Therefore, based on the above discussion, RNG is introduced to develop combined impact of population distribution and multi-CBD attraction on the topology of road network in this paper. We highlight the main contributions of our work at a glance below:

1. We develop the road network pattern framework considering population distribution and central business district based on the relative neighbor graph. For simplicity, we present our road network pattern model only with the consideration of the population distribution and central business district. However, the some other social-economic mechanisms, such as land use and environment, can readily be incorporate into our proposed model. This work adds to the body of knowledge in the road network pattern by considering the some social-economic phenomena.
2. Our numerical experiments demonstrate that our simulation results are very similar with the real network topology structure. Moreover, our model can be extended to the case of multi-CBDs. Results can provide useful insights in the urban road network planning.

The remainder of this paper is structured as follows. In section 2, we present the proposed model with explicit consideration of population distribution and CBD. The simulation results and the detailed analysis are presented in section 3. Two typical examples are presented to further demonstrate the effectiveness of the proposed model in Section 4. Section 5 concludes the paper and proposes the future research directions.

## 2. Model

In this section, we present a road network pattern model with the consideration of both population distribution and CBD. For simplicity, in Section 2.1, we list all notations used in this paper. The definitions of some of the variables are given in the subsequent sections when they are first used.

### 2.1. Notation

Consider a connected road network *G*(*V*,*A*), where *V* denotes the set of distinct communities and *A* represents the adjacent matrix of urban road network. The notations are adopted throughout this paper in Table 1.

**Table 1:** Notations.

Sets	Description
${\mathbb{R}}^{2}$	2-dimension Euclidean space
*p*_*i*_	the *i*-th distinct zone in ${\mathbb{R}}^{2}$, *i* = 1,2.,*n*
*q*	a zone in ${\mathbb{R}}^{2}$
*V*	the set of distinct zones, *V* = {*p*_1_,*p*_2_,…*p*_*n*_}
*d*(*p*,*q*)	the Euclidean distance between zones *p* and *q*
*x*_*i*_	the abscissa of the *i*-th zone in ${\mathbb{R}}^{2}$, *i* = 1,2.,*n*
*y*_*i*_	the ordinate of the *i*-th zone in ${\mathbb{R}}^{2}$, *i* = 1,2.,*n*
*U*_*pq*_	the utility between zones *p* and *q*
*H*_*p*_	the population of *p*-th zone
*L*_*pq*_	the Euclidean distance between zones *p* and *q*
*α*_1_	a positive polpulation-realtive parameter,
*α*_2_	a positive distance-realtive parameter
*m*_1_	a polpulation-realtive parameter
*m*_2_	a distance-realtive parameter

### 2.2. Relative neighborhood graph (RNG)

RNG was first introduced by Toussaint [33] in the computational geometry. RNG of a finite set *V* in the Euclidean space ${\mathbb{R}}^{m}$ is defined as an undirected graph with a set of distinct points *V* and a set of edges *RNG*(*V*) which are exactly those pairs (*p*,*q*) of points for which *d*(*p*,*q*) ≤ max_*z*∈*V*\{*p*,*q*}_{*d*(*p*,*z*),*d*(*q*,*z*)} [29, 33]. In fact, the minimum spanning tree is a subgraph of RNG, which implies that the network constructed according to RNG will have higher accessibility than that constructed according to MST. A comprehensive survey of RNG was provided in Jaromczyk and Toussaint [34] and Supowit [35].

How to find the RNG in a given set of distinct *n* zones in the Euclidean space? In fact, numerous research efforts focused on the algorithm of RNG in the three decades. For example, Supowit [35] developed how to construct the relative neighborhood graph efficiently in *O*(*n*log *n*) time. It can be computed in *O*(*n*) expected time for random set of zones distributed uniformly in the unit square [36]. The relative neighborhood graph can be computed in linear time from the Delaunay triangulation of the zone set. Therefore, according to the definition of RNG, The following is the procedure of RNG algorithm:

- **Step 1**. Calculating the distance for all pairs *d*(*p*_*i*_, *p*_*j*_), *i*, *j* = 1,…,*n*, *i* ≠ *j*.
- **Step 2**. For each pair (*p*_*i*_, *p*_*j*_), computing *d*^*k*^_max_ = max{*d*(*p*_*k*_, *p*_*i*_),*d*(*p*_*k*_, *p*_*j*_)}, *k* = 1,…,*n*, *k* ≠ *i*, *k* ≠ *j*.
- **Step 3**. For each pair (*p*_*i*_, *p*_*j*_), searching a *d*^*k*^_max_ which is smaller than *d*(*p*_*i*_, *p*_*j*_). If such a zone is not found, an edge is created between *p*_*i*_ and *p*_*j*_.

### 2.3. Utility of population distribution and CBD attraction

**Fig 1:**
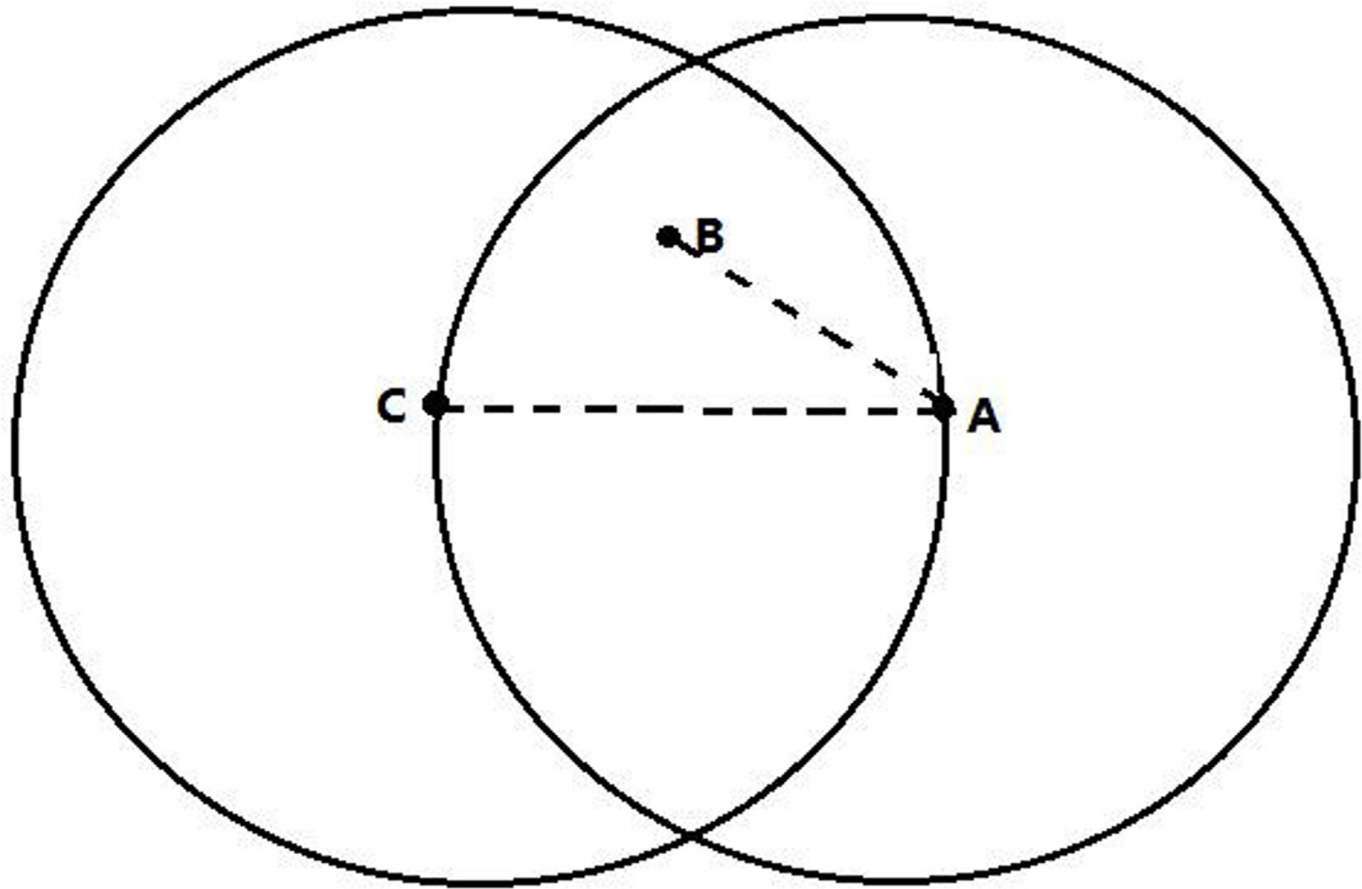
The uitility of population distribution.

In reality, population distribution has an influence on road network pattern. For example, the densely populated zones tend to have more convenient traffic conditions, thus these zones will have high accessibility. Therefore, in the road network pattern model, population distribution is an important factor. In this paper, it is assumed that the more populated zone is more likely to be connected to the new zone than less populated one. To facilitate the presentation of the essential ideas, Fig 1 clearly depicts the impact of population distribution. From this figure, we can see that the zone *A* is the relative neighbor of the zone *B* because there does not exist another zone closer to both *A* and *B*. According to the definition of RNG, zones *A* and *B* should be connected by a link directly. However, when the influence of the population distribution on the topology of road network is considered, that is *H*_*c*_ ≫ *H*_*B*_, the zone *A* is more likely to connected with the zone *C*.

**Fig 2:**
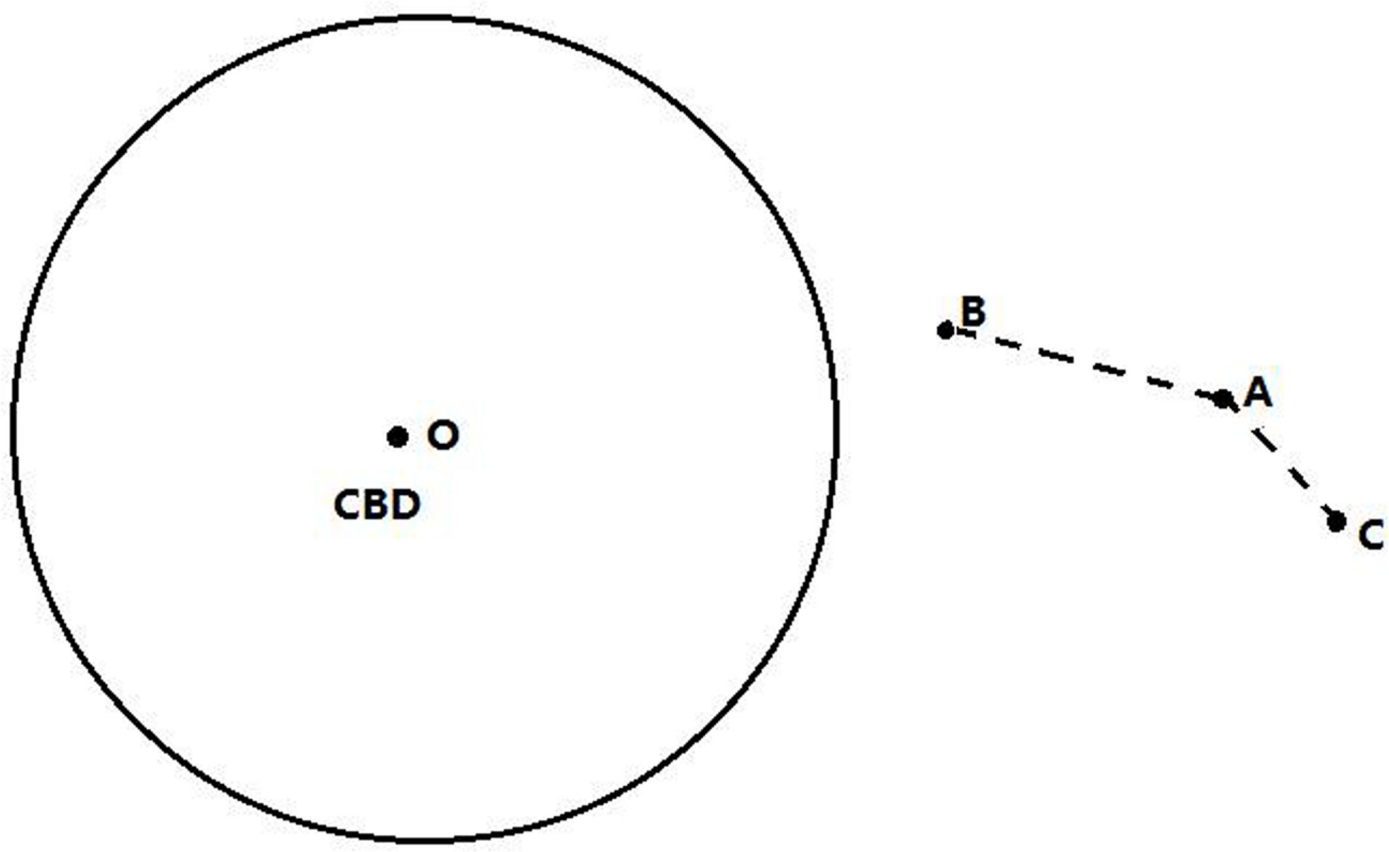
Attraction utility of CBD.

On the other hand, CBD is another important fact to consider in the study of road network pattern model because of the importance of CBD and its special geographical location. Therefore, in this paper, it is necessary to analyze the influence on the spatial structure of road network. It is natural to image that CBD should be equipped with convenient traffic condition. Similar to Fig 1, Fig 2 presents the influence of CBD attraction. As we can see, if the zone *A* is the relative neighbor of the zone *C*, the link between zones *A* and *C* should be built. However, if *L*_*OC*_ is much greater than *L*_*OB*_, it implies that the link between *A* and *B* should be built with a higher probability.

According to the above discussion, both the population distribution and the attraction of CBD should not be neglected in the study of road network spatial structure. Next, we investigate the combined influence of the population distribution and CBD attraction on the topology characteristics of road network. To this end, the utility is affected by the population distribution and CBD attraction and can be defined as follows:

$$U_{pq}=\alpha_{1}{\left(H_{p}+H_{q}\right)}^{m_{1}}-\alpha_{2}{\left(min(L_{Op}^{1}+L_{Oq}^{1},L_{Op}^{2}+L_{Oq}^{2},\ldots ,L_{Op}^{k}+L_{Oq}^{k})\right)}^{m_{2}},\alpha_{1}>0,\alpha_{2}>0$$ (1)

where *U*_*pq*_ and $L_{Op}^{1}$ denotes the utility between zones *p* and *q*, and the distance between zone *p* and the first CBD, respectively; *k* is the number of the CBD; and *α*_1_, *α*_2_, *m*_1_ and *m*_2_ are the parameters. The greater *α*_1_ and *m*_1_ indicate the greater impact of population distribution, while the greater *α*_2_ and *m*_2_ imply that the greater utility of CBD attraction. As we can see, Eq (1) has two components which, from left to right, represent the population utility and the CBD attraction, respectively. It is shown in Eq (1) that population distribution has a positive utility, and the distances between zones and CBD have a negative utility. Therefore, the notion of “relatively close” neighbors can be described as Eq (2), then *p* and *q* are “relatively close” neighbors if and only if:

$$d(p,q)-U_{pq}\leq \max(d(p,z)-U_{pz},d(q,z)-U_{qz}:z\in V-\{p,q\})$$ (2)

In the next section, we attempt to use the previous algorithm of RNG and the definition of utility in this section to simulate the road network growth.

## 3. Results and Discussion

In this section, a numerical simulated experiment is conducted to demonstrate the effectiveness of proposed model. To simplify the calculation, in our experiment, the number of CBD is assumed to be 2 and the parameters *m*_1_ and *m*_2_ is set to 1 respectively. In addition, the simulation results are obtained by the average of 100 experiments. In the following figures, the nodes’ colors and the links’ thick represent the population density and the traffic volume on the road network, respectively. In addition, the deeper color of nodes imply the higher population density.

### 3.1. The topology of road network

**Fig 3:**
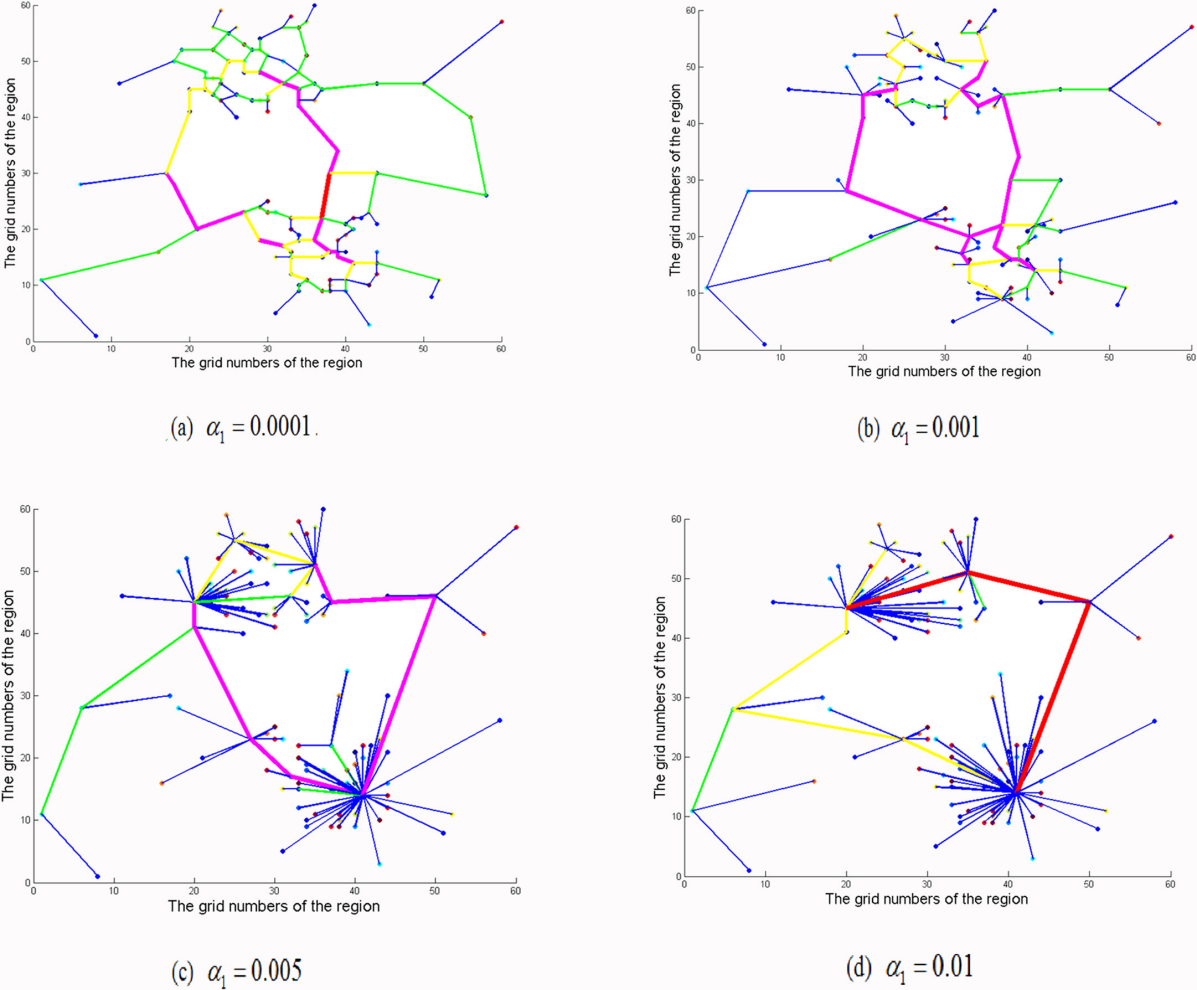
Topology of the road network considering only the population distribution.

To demonstrate the impact of population distribution and CBD attraction on the road network spatial structure, the topology of road network from either the aspect of population distribution or CBD attraction, or both is developed in this subsection. Fig 3 clearly depicts the topologies of road network with only the consideration of the population distribution when *α*_1_ = 0.0001, 0.001, 0.01 and 0.1, respectively. We can see that the topology of road network of gradually appears the radial network shape concentrate on the zones with the biggest population as the influence of population grows.

**Fig 4:**
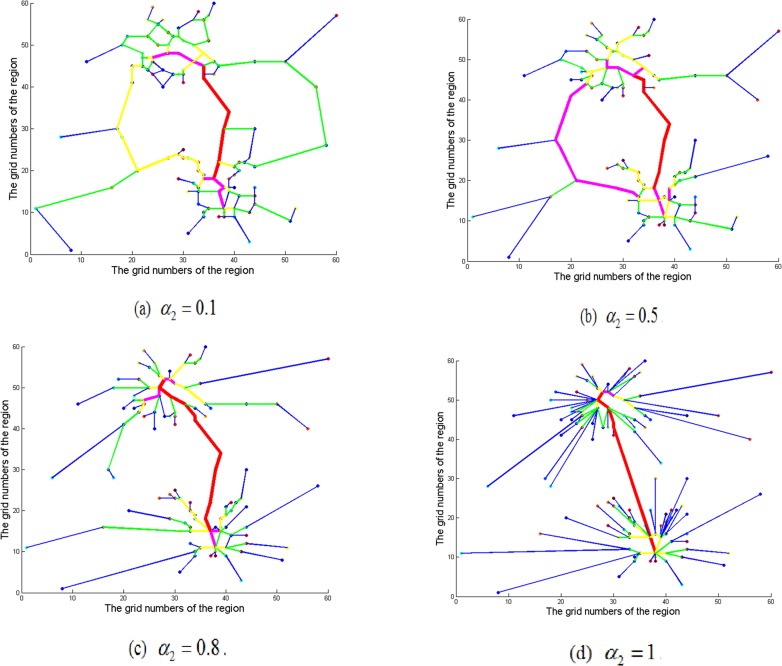
Topology of the road network considering only utility of CBD attraction.

To illustrate the impact of the topology of road network on CBD attraction, Fig 4 plots the topology of road network that only account for CBD attraction for *α*_2_ = 0.1, 0.5, 0.8 and 1, respectively. From Fig 4, it is clearly that the road network changes gradually from RNG to radial network with the increase of CBD attraction. A similar phenomenon can be observed from Fig 4. The topology of road network with only consideration of CBD attraction also displays the radial network. In addition, as shown in Fig 3(D) and Fig 4(D), when the parameters *α*_1_ and *α*_2_ become relatively large, the topology of road network shows the tendency of concentrating in some zones or CBDs overly.

**Fig 5:**
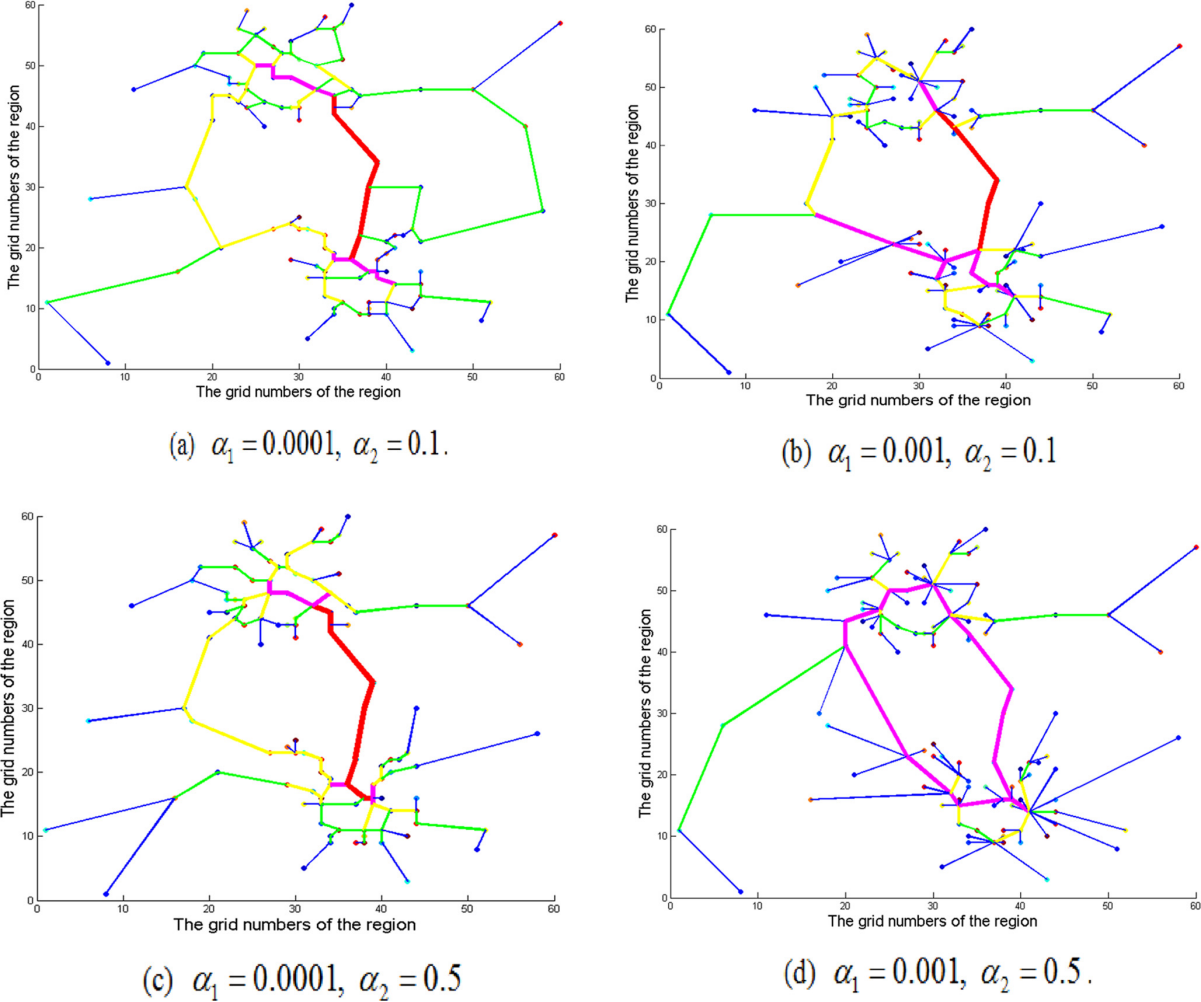
Topology of the road network considering jointly the population distribution and CBD attraction.

As can be seen from the previous discussion, both the population distribution and CBD attraction are the important factors that influence the topology of road network. To examine the impact of both population distribution and CBD attraction on the topology of road network, Fig 5 displays the results of the topology of the road network for the different *α*_1_ and *α*_2_. As expected, that the road network appears gradually radial shape as *α*_1_ and *α*_2_ grow. The difference between it and only consider the population distribution or CBD attraction is that the radial-network’s centers are not the zones with the most population or CBD, but somewhere in-between.

### 3.2. Circuitness and treeness

The basic structures of a planar transportation network can be divided into two categories: 1) circuit networks and 2) branching networks [34, 37]. Circuit networks are characterized by regional networks structured with closed circuits, where a circuit is a closed path (with no less than three links) with the same vertex as start and end. Branching networks are defined as tree structures with multiple connected links without any circuits. Specifically, a graph without cycles is referred as a forest and a connected forest is referred as a tree. Circuit and branching urban road network displays some typical connection patterns. In this paper, a circuit block is defined as a block that contains at least one circuit and contains neither bridges nor articulation points. A circuit block contains only one circuit and more than one circuit is called as a ring and web, respectively [37]. Note that ring and web are typical circuit networks; whilst star and hub-and-spoke are typical branching networks [37]. To examine the topology of road network from graph, the circuitness and treeness for a general network are defined as [14]:

$$\phi_{circuit}=\phi_{ring}+\phi_{web}$$ (3a)

$$\phi_{ring}=\frac{\text{Total length of links on rings}}{\text{Total length of links}}$$ (3b)

$$\phi_{web}=\frac{\text{Total length of links on webs}}{\text{Total length of links}}$$ (3c)

$$\phi_{tree}=1-\phi_{circuit}$$ (3d)

Clearly, these ratios vary from 0 to 1 and they indicate the extent to which the entire network is connected as circuits or trees. As we know, circuitness and treeness [37] are two important measures used to represent the topological properties of network, which reflect what extent the links of network are connected as circuits or trees. A high ratio of treeness implies a branching network structure, while a high ratio of circuitness indicates a circuit network. These two measures provide a consistent and computable way to examine typical topologies for the entire network based on digitized road networks [37]. Xie and Levinson [37] proposed an algorithm to determine whether a link is on the tree or on a circle for a given connected network, and this algorithm is adopted in this paper. The interested readers can refer to Xie and Levinson [37] for details on these results.

**Fig 6:**
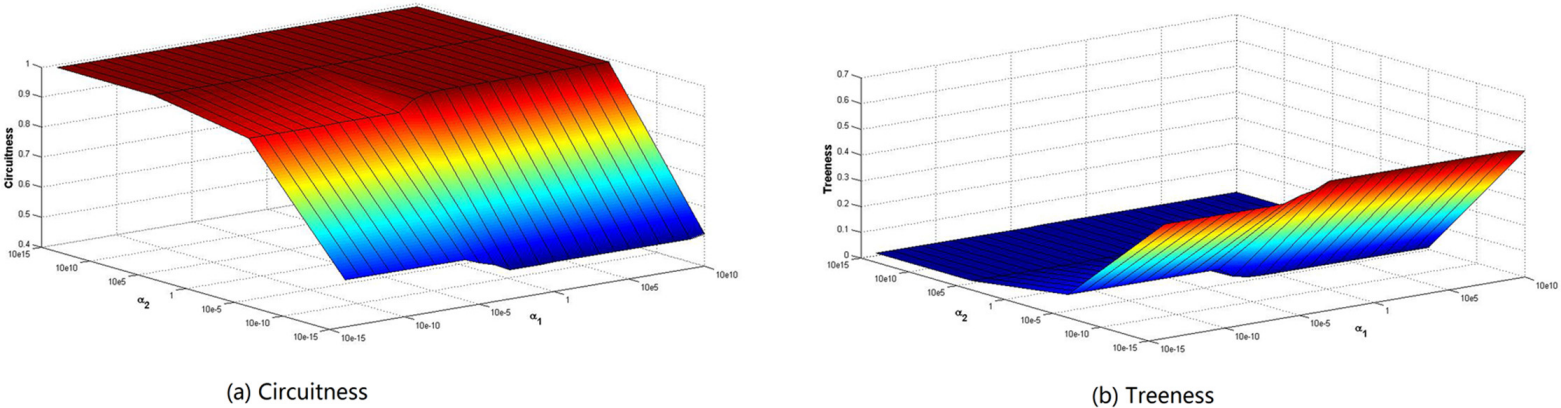
Circuitness and treeness of the road network.

To justify the impact of the population distribution and CBD attraction on circuitness and treeness, Fig 6 clearly depicts the sensitivity of the circuitness and treeness to the changes in the parameter *α*_1_ and *α*_2_ with jointly consideration of the population distribution and CBD attraction. As can be seen from Fig 6, when the parameter *α*_2_ ≥ 10^5^, circuitness and treeness almost remain unchanged for the parameter *α*_1_, while both circuitness and treeness are increasing trend with the parameter *α*_1_ when the parameter *α*_2_ < 10^5^. On the other hand, Circuitness and treeness is a monotonically increasing and deceasing function with respect to the parameter *α*_2_, respectively. It is clear that if one measure increases, and the other indexes will reduce since the sum of circuitness and treeness is equal to one. It is shown that the topology of road network changes between RNG and radial network with the change of the parameter *α*_1_ and *α*_2_.

### 3.3. Coverage

Coverage is also an important basic index for characterizing the topology property of the network, which represents the uniformity degree of the network [38]. A higher coverage implies that more grids are traversed by the link of network, and the topology of network is better.

To define the coverage of urban road network, we consider a lattice of equal length *y* on two sides which covers the entire network. Next, this lattice is divided into four equal parts each with length $\frac{y}{2}$ on both sides. Similarly, this lattice may be divided further. Then, the coverage can be defined as [34]:

$$Coverage(y_{i})=\frac{\ln(B(y_{i-1}))-\ln(B(y_{i}))}{\ln(y_{i})-\ln(y_{i-1})}$$ (4)

where *y*_*i*_ is the length of lattice after *i*-th subdivision, and *B*(*y*_*i*_) denote the number of lattices that the network links pass through at the *i*-th subdivision. This measure reflects the covering form of an urban road network. A greater coverage measure indicates that more lattices are passed by network links, and the covering form of the network is higher.

**Fig 7:**
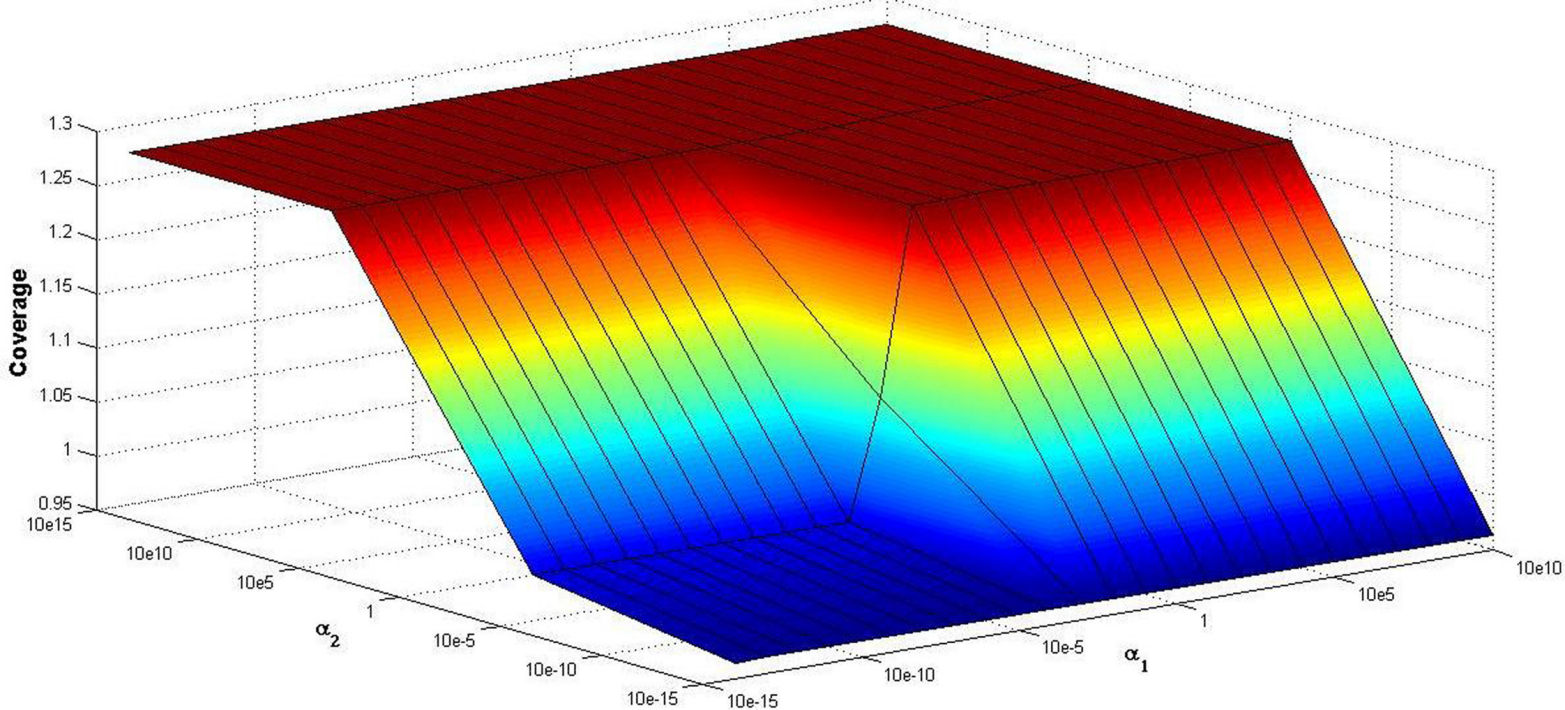
Coverage of the road network.

To demonstrate the uniformity of road network, we develop the coverage of the road network generated by our model in Fig 7. From Fig 7, it is clear that coverage rang from to 0.95 to 1.28. When the parameter *α*_1_ is fixed, coverage gradually increases as the parameter *α*_2_ grows. On the other hand, coverage is not always monotonously increased with the increase of the parameter *α*_1_. When *α*_2_ > 10^5^, coverage remain the same for the parameter *α*_1_, coverage shows a slowly rising trend when *α*_2_ < 10^5^.

### 3.4. Total length

**Fig 8:**
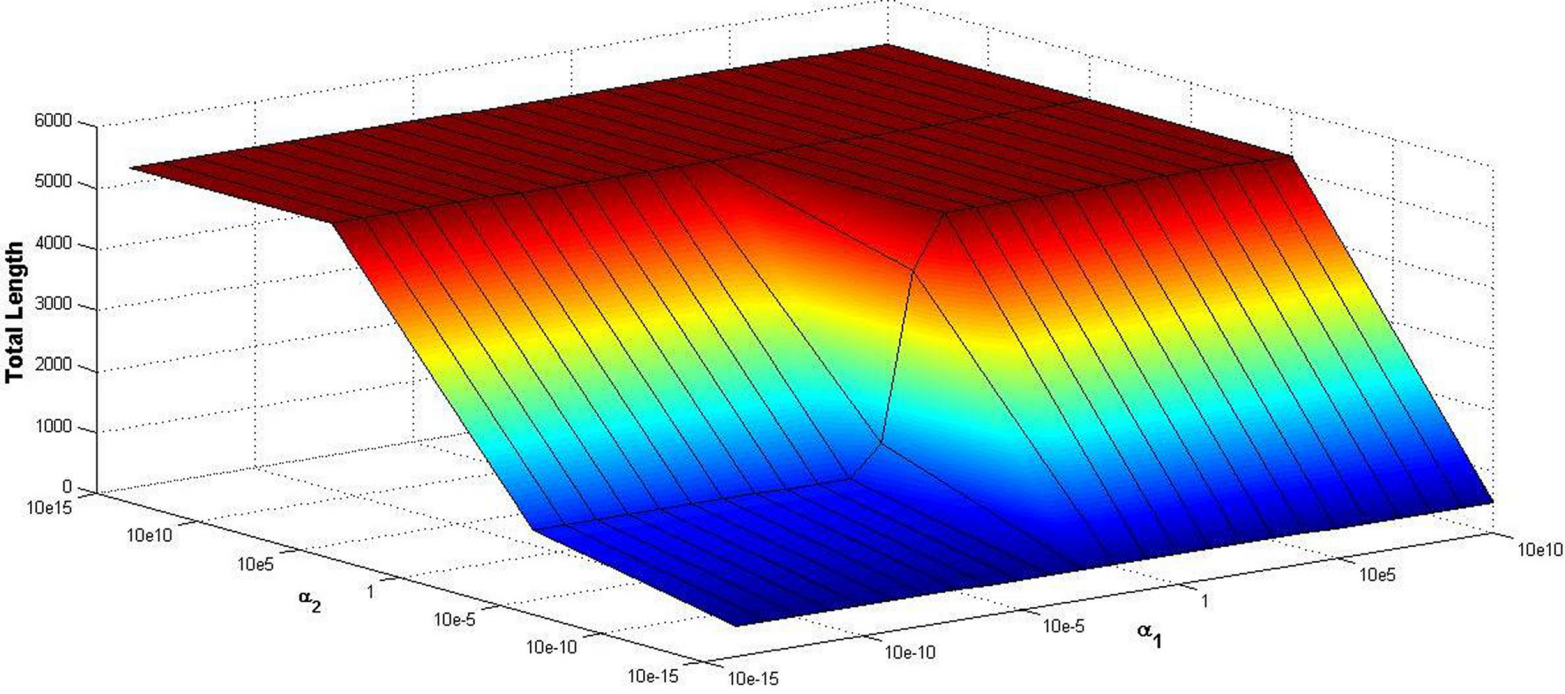
Total length of the road network.

Total length of road network is an important measure to judge whether the network topology is reasonable or not, which is defined as the sum of all the length of links on the road network. To illustrate the combined impact of the population distribution and CBD attraction on total length on road network, Fig 8 clearly depicts the total length of the road network generated by our model. As shown in Fig 8, the parameter *α*_1_ almost has no influence on total length when *α*_2_ ≥ 10^5^, while total length increases with the parameter *α*_1_ increases when *α*_2_ < 10^5^. On the other hand, it is obvious in Fig 8 that total length is an increasing function with respect to the parameter *α*_2_ for a fixed parameter *α*_1_. It is continuing to show that it has different utilities between population distribution and CBD attraction. Therefore, we can obtain the different topologies by adjusting the parameters *α*_1_ and *α*_2_.

## 4. Typical Examples

**Fig 9:**
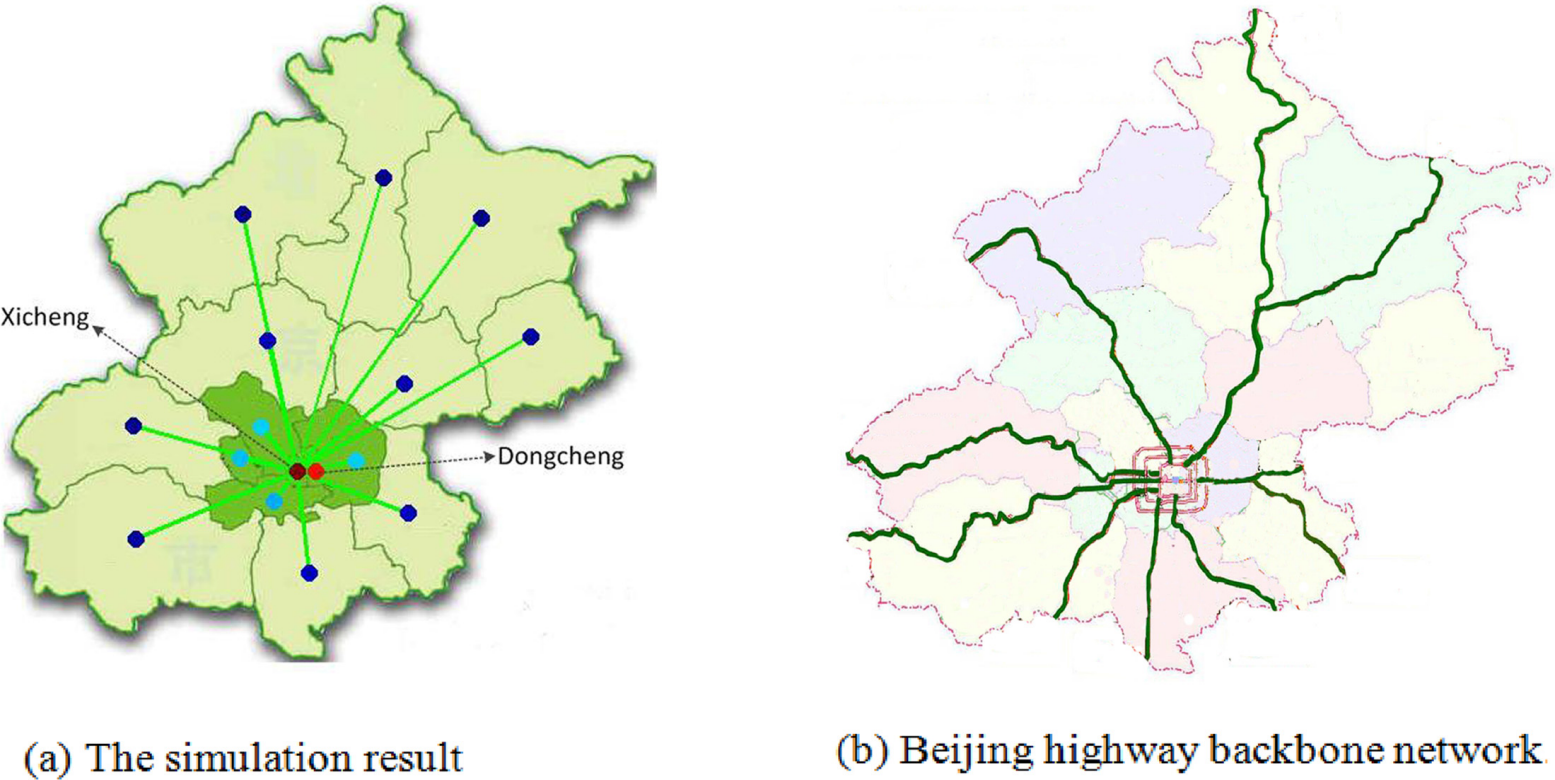
Comparison between the simulation result and real backbone network.

**Fig 10:**
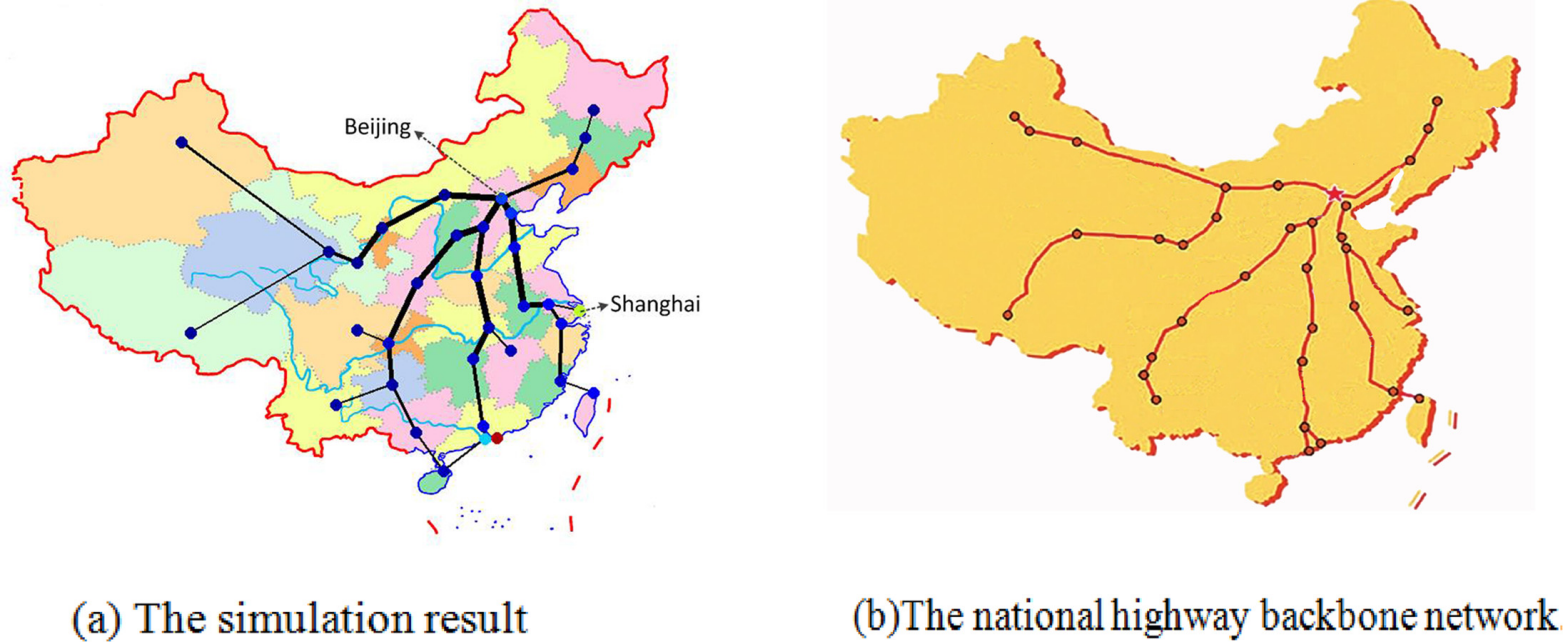
Comparison between the simulation result and real backbone network.

**Fig 11:**
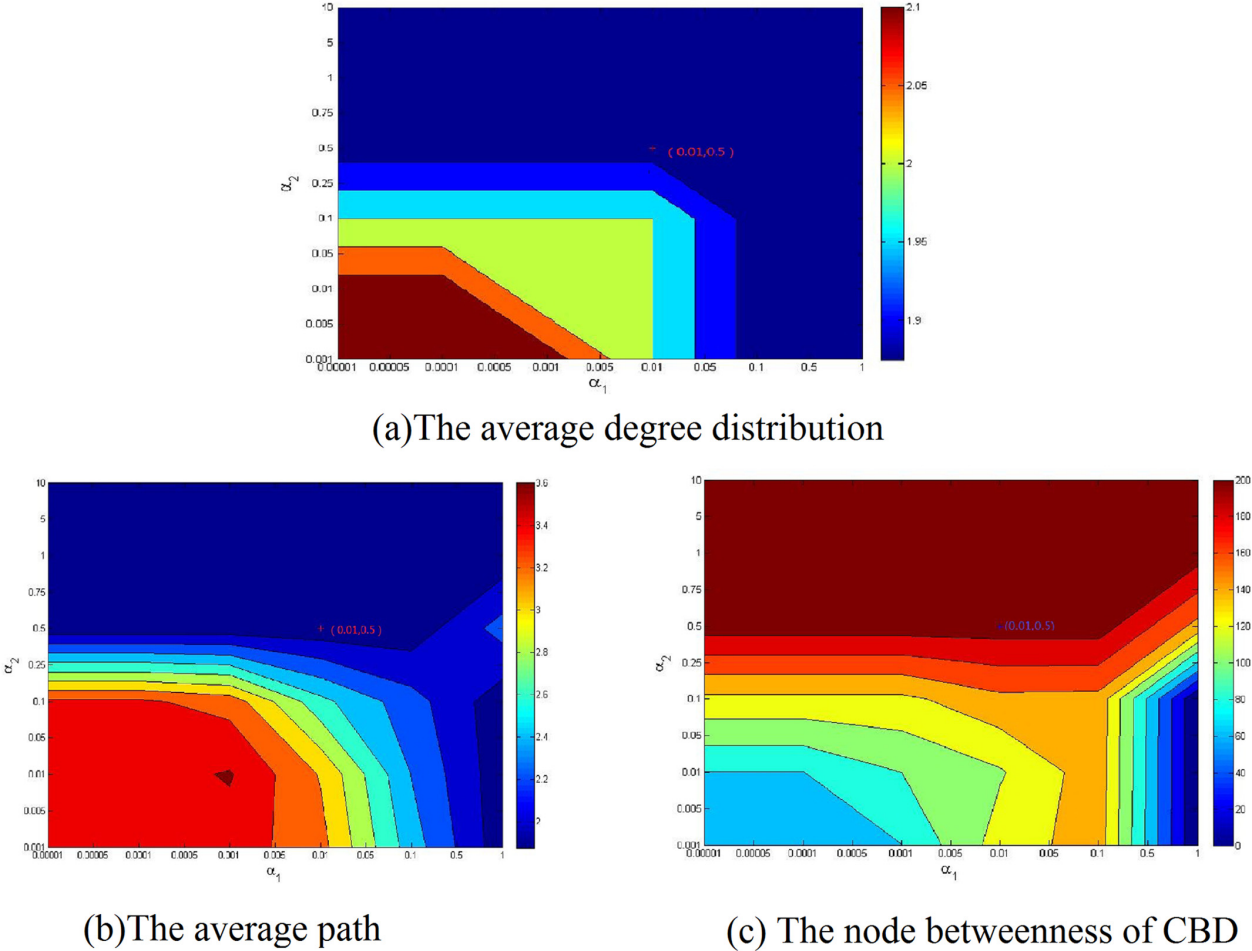
Average degree distribution, average path lengths and node betweenness of CBD of simulated Beijing road network under various combinations of the parameters *α*_1_ and *α*_2_.

**Fig 12:**
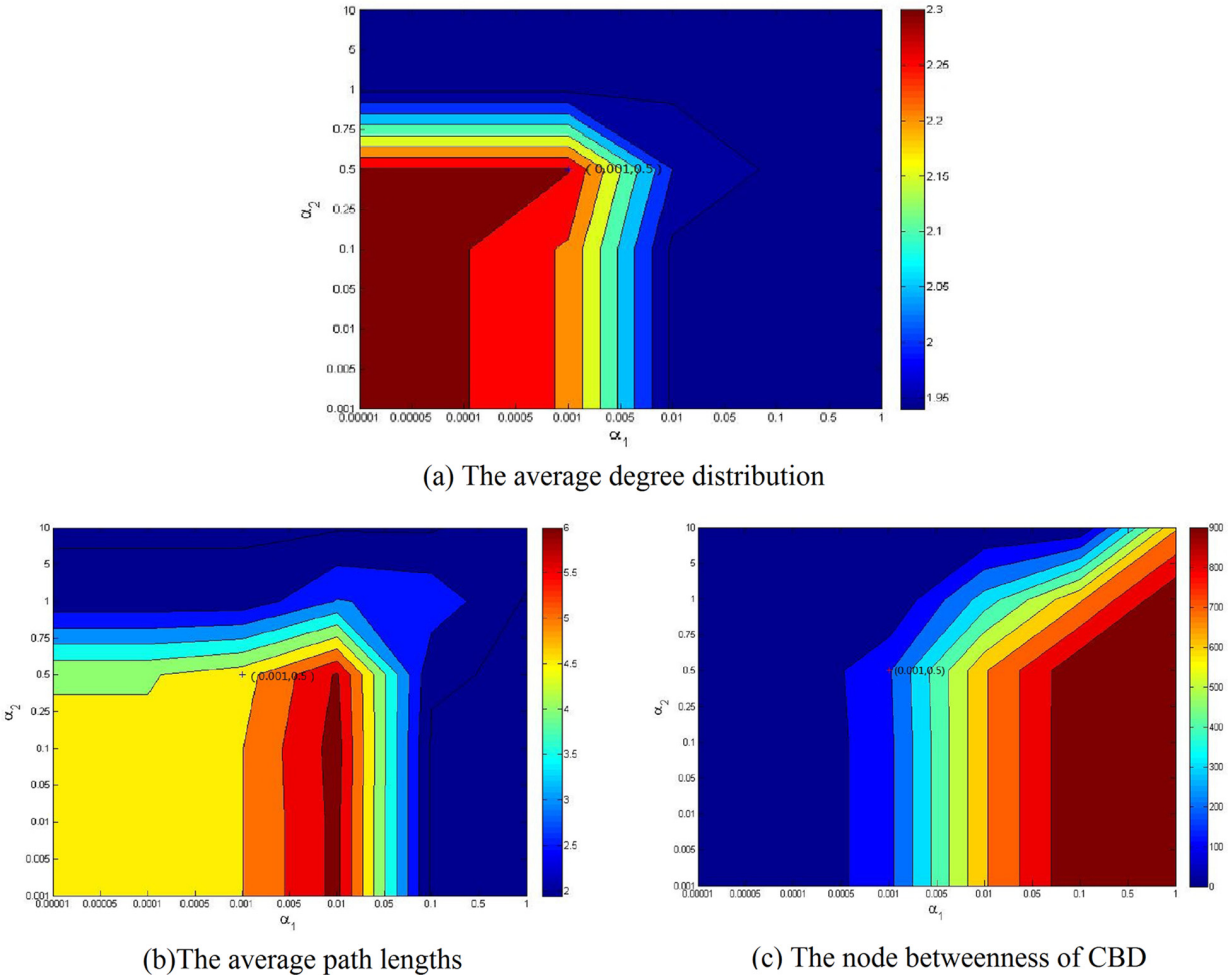
Average degree distribution, average path lengths and node betweenness of CBD of simulated China road network under various combinations of the parameters *α*_1_ and *α*_2_.

As discussed above, the population distribution and CBD are two important factors that affect the topology of road network. To illustrate our model, the simulation experiments based on the real road network are implemented in this section. To this end, we will simulate the topologies of Beijing and China in this section. For the given population and CBD of the Beijing and China, districts and cities are viewed as nodes respectively, while highways represent the links, road network topology can be simulated with respect to changing parameters *α*_1_ and *α*_2_. How to choose the parameters *α*_1_ and *α*_2_? We consider calibrating them from two aspects. One the one hand, the choice of the parameters to make the topology of the simulation network is similar with the real network. Fig 9 provides the comparison results between the simulation result and Beijing highway backbone network, where Dongcheng and Xicheng districts are set to the two CBD and the parameters *α*_1_ and *α*_2_ is set to be 0.01 and 0.5, respectively. In Fig 9, the colors of nodes denote its population; and the deeper color of the node implies the more population. Fig 10 displays the comparison results between the simulation and the national highway backbone network, where Beijing is viewed as only one CBD with *α*_1_ = 0.001 and *α*_2_ = 0.5. Fig 9 and Fig 10 display that the simulation result is very analogue to the real highway backbone network. Fig 11 shows that the average degree distribution, average path lengths and node betweenness of CBD of the simulated Beijing road network are similar with the real Beijing road network when the parameter *α*_1_ = 0.01 and *α*_2_ = 0.5. Fig 12 gives the average degree distribution, average path lengths and node betweenness of CBD of the simulated China road network. It can be seen that these measures of the simulated and real China road network is similar when the parameters *α*_1_ = 0.001 and *α*_2_ = 0.5.

Table 2 describes the measures of simulated and real road network of Beijing when the *α*_1_ = 0.01 and *α*_2_ = 0.5, while Table 3 reports the comparison results for *α*_1_ = 0.01 and *α*_2_ = 0.5. As can be seen, these measures are very close and the relative error is no more than 15%. It is continuing to shows the simulated and real network is very analog.

**Table 2:** The comparison results of real and simulated network of Beijing.

Beijing(Dongcheng and Xicheng)	Real network	Simulated network	Relative error
Average degree distribution	1.8750	1.750	6.67%
Average path length	1.8750	2	6.25%
Node betweenness of CBD	210	189	10%

**Table 3:** The comparison results of real and simulated network of China.

China(Beijing)	Real network	Simulated network	Relative error
Average degree distribution	1.9394	1.9394	0%
Average path length	5.7538	6.197	7.15%
Node betweenness of CBD	609	702	13.25%

**Fig 13:**
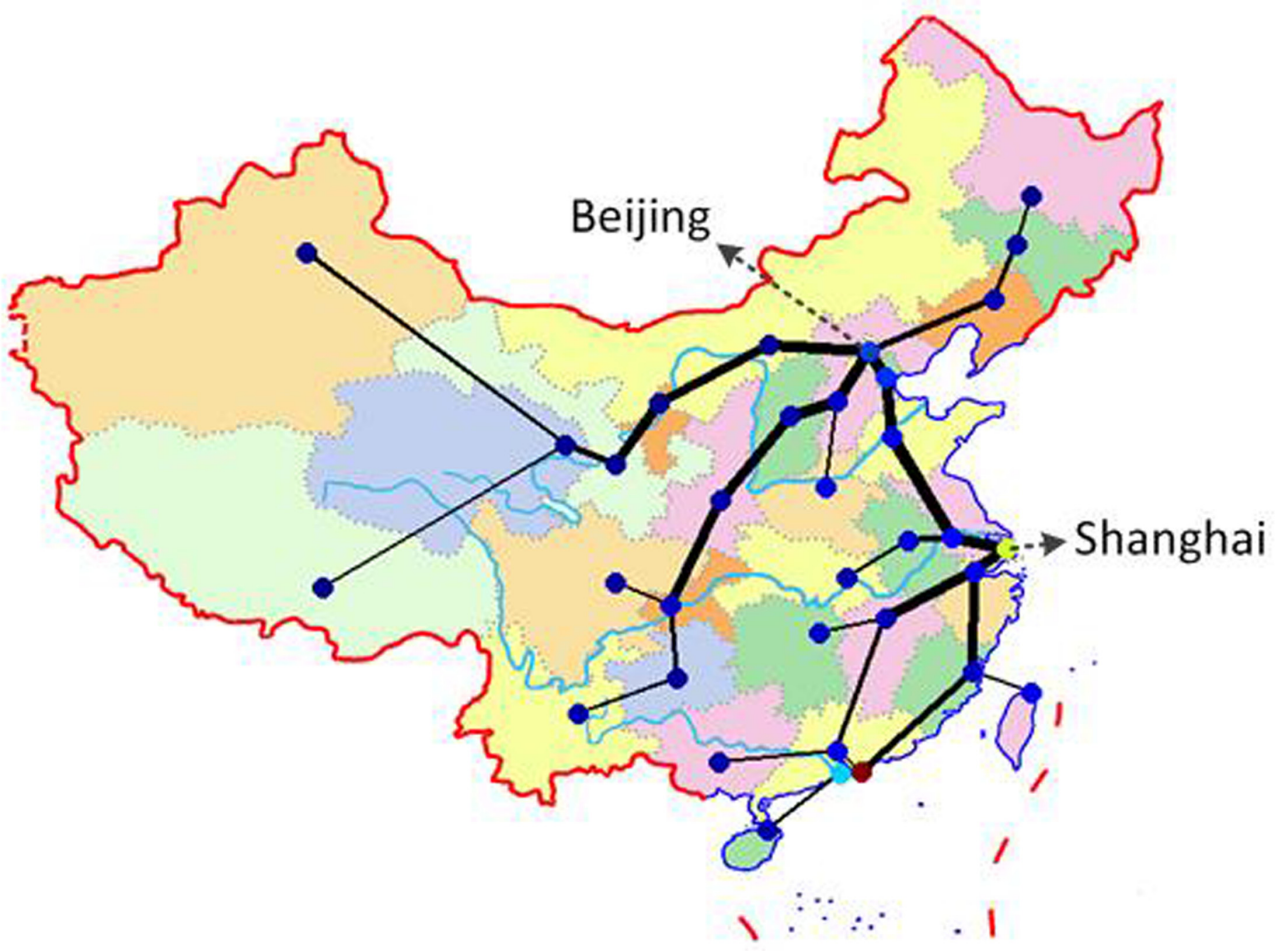
Road network topology of China (Beijing and Shanghai as its CBDs).

With the development of economic, the new CBDs may be appearing. Fig 13 gives the topology structure of road network with multi-CBDs. We can see that the backbone topology of road network in China if we assume that there are the two CBDs such as Beijing and Shanghai in the network. It can be seen from Fig 13 that the degrees of Shanghai increase, while the degrees of Beijing reduce. This shows that the two center simulation network has the better diffluent function than the one center network. Moreover, we also compare the simulation results for one center and two centers in Table 4, where zero in Table 4 implies no shortest paths passing through Shanghai. Clearly, the case with two centers will have a more diffluence roles.

**Table 4:** The comparison simulated results of one CBD and two CBDs of China.

	China(Beijing and Shanghai)	China(Beijing)
Average degree distribution	2	1.9394
Average path length	6.4510	6.197
Node betweenness of CBD(Beijing)	724	702
Node betweenness of CBD(Shanghai)	460	0

## 5. Conclusions

This paper developed a road network pattern model. In the model, the impact of population distribution and CBD attraction on the topology of road network is simultaneously considered. The relative neighbor graph (RNG) is introduced as the link connecting mechanism in our model. A simulation experiment is conducted to demonstrate the performance of proposed model. Moreover, some measures, such as coverage, circuitness, treeness, and total length, are introduced to illustrate the impact of population distribution and CBD attraction on the topology of road network. Finally, our proposed model is used to simulate the road network of Beijing and China. The simulation results demonstrate the topology of the road network is similar to the real situation.

There are some possible further research directions. First, the travelers’ route choice behavior and the limit financial budget during the process of building the road is not account for in this paper. One possible extension is to incorporate them into the proposed framework in future studies. Secondly, the some other social-economic mechanisms, such as land use and environment, can be introduced into the proposed framework.
